# *Staphylococcus capitis*: Review of Its Role in Infections and Outbreaks

**DOI:** 10.3390/antibiotics12040669

**Published:** 2023-03-29

**Authors:** Victoria Heath, Elaine Cloutman-Green, Samuel Watkin, Magdalena Karlikowska, Derren Ready, James Hatcher, Nicola Pearce-Smith, Colin Brown, Alicia Demirjian

**Affiliations:** 1 Department of Microbiology, Virology and Infection Control, Great Ormond Street Hospital, London WC1N 3JH, UK; 2 National Institute of Health Research Great Ormond Street Hospital Biomedical Research Centre, London WC1N 1EH, UK; 3 Healthy Infrastructure Research Group, University College London, London WC1E 6BT, UK; 4 United Kingdom Health Security Agency, London SW1P 3JR, UK; 5 Health Protection Research Unit in Behavioural Science and Evaluation, University of Bristol, Bristol BS8 1QU, UK; 6 Department of Paediatric Infectious Diseases & Immunology, Evelina London Children’s Hospital, London SE1 7EH, UK; 7 Faculty of Life Sciences & Medicine, King’s College London, London WC2R 2LS, UK; 8 Health Protection Research Unit, Imperial College London, London SW7 2BX, UK

**Keywords:** *Staphylococcus capitis*, *S capitis*, NRCS-A, neonatal intensive care unit, biofilm, environment, decontamination, vancomycin, antibiotic resistance

## Abstract

In June 2021, a national incident team was formed due to an increased detection of *Staphylococcus capitis* in samples from hospitalised infants. *Staphylococcus capitis* has been known to cause outbreaks in neonatal units across the globe, but the extent of the UK spread was unclear. A literature review was undertaken to support case identification, clinical management and environmental infection control. A literature search was undertaken on multiple databases from inception to 24 May 2021, using keywords such as “*Staphylococcus capitis*”, “NRCS-A”, “*S. capitis*”, “neonate”, “newborn” and “neonatal intensive care unit” (NICU). After screening, 223 articles of relevance were included. Results show incidences of *S. capitis* outbreaks have frequently been associated with the outbreak clone (NRCS-A) and environmental sources. The NRCS-A harbours a multidrug resistance profile that includes resistance to beta-lactam antibiotics and aminoglycosides, with several papers noting resistance or heteroresistance to vancomycin. The NRCS-A clone also harbours a novel *SCCmec-SCCcad/ars/cop* composite island and increased vancomycin resistance. The *S. capitis* NRCS-A clone has been detected for decades, but the reasons for the potentially increased frequency are unclear, as are the most effective interventions to manage outbreaks associated with this clone. This supports the need for improvements in environmental control and decontamination strategies to prevent transmission.

## 1. Introduction

A frequent cause of morbidity and mortality in hospitalised infants, particularly in those with very low birth weights, is late-onset neonatal sepsis. The most frequent cause of late-onset sepsis is coagulase-negative staphylococci, including *Staphylococcus capitis*. One particular clone of *S. capitis*, the NCRS-A clone, demonstrates resistance to aminoglycosides (e.g., gentamicin) and beta-lactam antibiotics, which are agents frequently used in empiric treatment of late-onset neonatal sepsis. The UK Health Security Agency noted a possible rise in *S. capitis*-related invasive infections in hospitalised infants during the summer of 2020. In response, a national incident was set up, and a review of the literature was carried out.

## 2. Methods

We performed literature searches in Ovid Medline, Embase and EmCare, the Cochrane Library, Web of Science, Science Direct and Google Scholar, from database inception to 24 May 2021. Abstracts (*n* = 56) and book chapters (*n* = 28) were excluded, along with articles that were unavailable (*n* = 7) or in non-English languages (*n* = 31).

Of the remaining records (*n* = 5770, full articles were screened for inclusion. Full-text records were excluded if they were not primary articles specific to *S. capitis*, did not discuss *S. capitis* in the text of the articles (*n* = 244), or did not cover clinical or human infection (*n* = 50). This included *S. capitis* papers that focused on veterinary care (*n* = 33) or other nonhealthcare settings, such as the environmental industry.

## 3. Results

After screening and exclusion, there were 223 studies remaining. The findings of the 223 studies were thematically grouped and are presented below (Table 1). The majority of the papers focussed on antimicrobial resistance of *S. capitis* (*n* = 79), with 67 papers covering clinical management in both adults (*n* = 37) and neonates (*n* = 30).

## 4. Characterisation of *S. capitis*

*S. capitis* was first isolated from human skin in 1975 [1]. Subsequently, it has been differentiated into two new subspecies: *S. capitis* subspecies *capitis* and *S. capitis* subspecies *ureolyticus*. The main differentiating feature between the two is the ability of *S. capitis* subspecies *ureolyticus* to produce urease, to produce acid aerobically from maltose and its fatty acid profile [2,3].

When undertaking phenotypic-based identification, *S. capitis* isolates can be distinguished from other coagulase-negative staphylococci (CoNS) by their large colony size and colonial appearance when incubated in moist conditions for 5 days at 37 °C on MRSA Brilliance 2 agar (Oxoid^®^, Basingstoke, UK). *S. capitis* NRCS-A isolates grow as mauve colonies with a cream colour halo, whereas MRSA isolates form blue colonies and CoNS form small white colonies [4]. Anecdotal reports from colleagues working in this field have, however, highlighted challenges with this identification method.

When comparing molecular and phenotypic identification techniques, Pennington et al. [5] demonstrated 90% similarity between *S. capitis* and *S. epidermidis* using Sodium Dodecyl Sulfate–Polyacrylamide Gel Electrophoresis (SDS PAGE) analysis. The authors further illustrated that the standard biochemical test panel in API Staph (API Laboratory Products, Canada) was unable to robustly differentiate between the species, and this level of similarity led to some reports suggesting that *S. capitis* similarity with *S. hominis* and *S. warneri* meant that they have been overclassified into too many species [5]. Enhanced characterisation therefore required rRNA gene restriction patterns [6]. This was confirmed in work undertaken by Carretto et al. [7], which demonstrated that ribotyping was more accurate than API 20 Staph (bioMérieux, Lyon, France) identification but that the API 20 was more cost efficient, meaning that enhanced identification led to a cost increase. Other phenotypic systems, such as Vitek (bioMérieux, Lyon, France), have been evaluated to successfully identify 67% of CoNS isolates, compared with 61% for API 20GP (bioMérieux, Lyon, France) [7].

More recently, molecular tools, such as real-time PCR, and phenotypic tools, such as Matrix-Assisted Laser Desorption/Ionisation-Time of Flight Mass Spectrometry (MALDI-ToF), have increased in availability and demonstrated potential for rapid identification of isolates. RT-PCR offers faster identification than conventional PCR; however, mixed bacterial cultures may decrease assay reliability [8]. A recent study compared molecular and phenotypic methods for identification of 134 CoNS, including 10 *S. capitis* isolates. RT-PCR with a *tuf* gene target and MALDI-ToF each identified all 134 correctly: Vitek identified 121 (90.3%) and biochemical phenotyping testing 103 (76.8%). The 10 *S. capitis* isolates were misidentified by both the Vitek and biochemical characterisations [9]. Accurate species identification via *tuf* gene PCR and MALDI-ToF was also demonstrated by Carpaij et al. [10], with the techniques being 100% congruent for 93 CoNS isolates, of which 13 were *S. capitis* [10].

Other molecular techniques requiring sequencing post-target amplification have been evaluated for staphylococcal species differentiation. Spanu et al. [11] demonstrated that PCR of *rpoB* gene provided correct identification of 332/335 CoNS isolates [11]. Enterobacterial Repetitive Intergenic Consensus (ERIC) PCR was validated for 200 CoNS species, of which 17 were *S. capitis*, 14 were subspecies of *ureolyticus* and 3 were subspecies of *capitis*. Enterobacterial Repetitive Intergenic Consensus (ERIC) PCR had low discriminatory power, except for *S. hominis*, and was not able to distinguish between subspecies of *S. capitis*, where all 17 isolates were considered clonal [12].

A study by Song et al. [13] demonstrated that 16S rRNA sequencing could not distinguish between *S. capitis* and *S. caprae*, leading to concern about the method for CoNS characterisation [13]. Ederveen et al. [14] further demonstrated that *S. epidermidis* and *S. capitis* cannot be distinguished between either the V1-V2 or V3-V4 16S subregions, and therefore, species identification for *S. capitis* utilising 16S rRNA sequencing should be interpreted with caution [14]. High-resolution melt analysis was utilised by Slany et al. [15] to analyse melt profiles of the 16S rRNA gene to identify staphylococcal species and showed a single nucleotide polymorphism change in *S. capitis*, meaning it was possible to differentiate; however, it was considered an epidemiological rather than an identification technique [15].

## 5. Pathogenesis

Proteins identified in certain strains of *S. capitis* are believed to be required for functions including virulence, adherence and biofilm formation [16]. However, the *S. capitis* genome does not have the same variety of exotoxins that are found in *Staphylococcus aureus* [17].

In terms of colonisation, the *S. capitis* TE8 strain has a number of genes postulated to play an important role in adherence and skin colonisation. The genome contains both the *ica*ADBC gene for intracellular adhesion and the *icaR* regulatory gene. Kumar et al. [16] report that the *S. capitis* TE8 strain contains 14 adhesins to improve adherence, and they support the effectiveness of the strain in colonising human skin [16].

In a study by Brandi et al. [18], *S. capitis* isolates demonstrated the strongest urease activity of all non-*H. pylori* bacteria isolated from the stomach of patients. *S. capitis* subspecies *ureolyticus* were able to survive for at least an hour in stomach acid pH conditions, exhibiting greater resistance than *Helicobacter pylori*, supporting colonisation of the digestive system [18]. Despite these pathogenicity factors, when invasion studies have been undertaken in HeLa cells, *S. capitis* isolates exhibited lower invasive ability than *S. auricularis* (1–10%); however, all bloodstream isolates included in this study were clonally related and therefore may not be representative of other strains [19].

## 6. Biofilm

The primary pathogenicity factor identified for *S. capitis* species was the ability to form biofilm. The percentage of *S. capitis* strains that were able to produce biofilm varied greatly between studies. Szczuka et al. [19] recorded that 87.5% of *S. capitis* strains demonstrated the ability to form a biofilm [19]. In contrast, Koksal et al. [20] recorded only 7% of *S. capitis* strains isolated from bacteraemia patients in Turkey as biofilm-positive, compared with 71% in *S. epidermis* and 43% across all CoNS strains [20]. The relative level of biofilm production may be lower in some *S. capitis* strains compared with those of other CoNS. A comparison of *S. capitis* AYP1020 with *S. epidermidis* RP62a demonstrated a six-fold decrease in biofilm production in the *S. capitis* strain [17]. The presence of the *ica* operon has been demonstrated to play a key role in biofilm production in *S. capitis*, as well as in other CoNS, with all biofilm-producing species identified as *ica*-gene-positive [19].

The ability of *S. capitis* to form biofilm has been linked with particular experimental conditions and environmental cues, for example, growth in media with high osmolarity [21]. Detection of biofilm production has therefore proved complex in some strains and required provision of specific induction conditions. One study demonstrated that 4/5 *S. capitis* strains produced higher density biofilms when platelet concentrates were present [22]. In another study, the presence of trypsin (serine protease) was demonstrated as necessary to induce biofilm formation [23].

Antibiotic-resistant phenotypes have been frequently associated with biofilm-producing phenotypes, with Koksal et al. [20] determining that methicillin resistance was significantly higher in biofilm-positive strains (81%) compared with negative strains (57%) [20]. Kitti et al. [24] also noted a correlation between biofilm-associated genes and biofilm production in all multidrug-resistant CoNS, including *S. capitis* [24]. In erythromycin-resistant strains, Cui et al. [25] demonstrated that biofilm density increased when exposed to erythromycin but not erythromycin-sensitive strains, leading to antibiotic exposure being considered as another factor in biofilm formation [25]. In another study, oxacillin-stimulated biofilm growth was demonstrated in two *S. capitis* strains that had been previously categorised as biofilm nonproducers, although no specific mechanism was identified [26]. Furthermore, rifampicin therapy was linked to rifampicin resistance genome changes and found to promote increased bacterial growth rate, biofilm formation and increased ability to survive in whole blood [27]. Therefore, the interaction between resistance and biofilm production is likely to be bidirectional.

## 7. Antimicrobial Resistance

In addition to the antimicrobial-resistant phenotypes linked to biofilm production, *S. capitis* holds a number of genetic determinants of resistance, which can make infections recalcitrant to therapy.

There are published data identifying plasmids or mobile genetic elements that encode for multidrug resistance in *S. capitis*. *S. capitis* isolates are frequently associated with SCC*mec* mobile genetic elements, which are a vehicle for exchanging resistance genes between staphylococcal species and are widely distributed among CoNS and *Staphylococcus aureus* [28]. The NRCS-A strain is associated with an SCC*mec* cassette, which is structurally similar to the type V SCC*mec* resistance cassette that has been characterised as providing resistance to β -lactams and demonstrating reduced susceptibility to aminoglycosides and vancomycin [17,29]. NRCS-A clones are associated with a novel SCCmec-SCCcad/ars/cop composite island, which has likely emerged from two independent acquisition events [30].

Vancomycin is a frequently utilised antimicrobial in *S. capitis* infection, but treatment failures have frequently been associated with vancomycin heteroresistance, leading to scenarios such as persistent bacteraemia [31]. Although vancomycin heteroresistance has been noted in a number of clinical CoNS isolates; it is most commonly present in *S. capitis*, particularly the NRCS-A clone, where it leads to the acquisition of vancomycin resistance at a 1.9-fold faster rate than the control *S. aureus* Mu3 strain [31]. The type-V-related staphylococcal cassette chromosome *mec* (SCC*mec*) associated with the NRCS-A clone leads to reduced susceptibility or resistance, resulting in significantly reduced management options, especially within the NICU setting [32].

For invasive infections caused by vancomycin-resistant strains of *S. capitis*, linezolid is an important therapeutic option. Resistance is primarily derived from point mutations in domain V of the 23S rRNA drug target site (G2576T, C2104T, G2447T, C2561Y, C2131T, G2576U and G2603T) and/or the presence of *cfr* (chloramphenicol–florfenicol resistance) genes, which encode for a 23S rRNA methyltransferase that confers resistance to linezolid [33]. Within patients whose isolates demonstrated linezolid resistance, all had prior exposure to linezolid therapy [34,35]. All of the linezolid-resistant isolates were vancomycin-sensitive, although some were resistant to both methicillin and aminoglycosides [36,37,38]. Induction of linezolid resistance within the NRCS-A clone should therefore be considered when making patient management decisions.

Oxacillin resistance in *S. capitis* is common, with between 27.2% and 86% of isolates containing the *mecA* gene [39]. Resistance to other antimicrobials have been reported from retrospective isolate studies, including telavancin [40], the bacteriocin nisin [41], tetracycline [42] and daptomycin [43]. Other resistance mechanisms associated with *S. capitis* include the multidrug-resistant plasmid pSC16875, which leads to isolates that are not only resistant for fusidic acid but are also positive for the *qacA* gene, which encodes for an efflux pump which can extrude chlorhexidine [44].

The NRCS-A clone has also been found to harbour the *nsr* gene, which confers nisin resistance. The nisin-resistant phenotype of the NRCS-A *S. capitis* clone is thought to impact the establishment of the neonatal gut microbiota following birth, as it is a bacteriocin with activity against other Gram-positive microorganisms [41].

Novel antimicrobials are being investigated which have demonstrated activity against *S. capitis*, including apolipophorin III, an insect-derived protein with moderate activity [45]; nemonoxacin, a nonfluorinated quinolone which has demonstrated broad-spectrum activity [46]; and Lausporin, a short cationic amphipathic molecule [47].

## 8. Clinical Presentations and Management

Within adults, *S. capitis* infection has resulted in a number of different clinical presentations. These include the following:

- Native valve endocarditis [48,49,50,51,52];
- Prosthetic valve endocarditis [53,54,55,56,57,58];
- Osteomyelitis [59,60,61,62];
- Polymicrobial infections [63,64,65,66];
- Surgical implant infections [67,68,69,70,71];

Other conditions have included case reports of pyomyositis, peritonitis, septic arthritis and community-acquired meningitis. [72,73,74,75].

A variety of antimicrobial treatment options were used in conjunction with surgical interventions and removal of implants/prosthetic devices where appropriate. For endocarditis, vancomycin plus gentamicin in the presence of a prosthetic valve or rifampicin with a native valve was common [50,51,52,54,55,56,57,58]. Other commonly used antibiotics have included meropenem and clindamycin in implant/device-related infections [67,68,69,70,71]. With the exception of cases of native valve endocarditis (*n* = 4), where 50% of patients died (*n* = 2) and the remaining had long-term complications (*n* = 2) [50,51,52], *S. capitis* infection in other sites resolved using the described management.

Within the paediatric and neonatal population, the main presentation of *S. capitis* infection is neonatal sepsis (see Section 9). Neonates with *S. capitis* infection often have previous exposure to ß-lactam and aminoglycoside antibiotics [76]. Invasive infection due to *S. capitis* in these patients is frequently managed using either vancomycin or linezolid [77]. In this patient population, *S. capitis* can cause non-catheter-related sepsis, which is hypothesised to be related to translocation of gut microbiota rather than invasion through the catheter as port of entry [78].

## 9. *Staphylococcus capitis* Infection and Colonisation in Neonates

Skin colonisation is well-described in *S. capitis*, and skin was the location of the first described isolation [1]. In one of the earlier studies, Savey et al. [79] identified 256 staphylococcus species from 10 neonates in 1989, of which 0.4% were *S. capitis*, all present on the soles of their feet [79]. In an intestinal colonisation study undertaken by Cossey et al. [80], out of 1045 faecal samples collected from 150 neonates, 11% were colonised with Staphylococcus species by the 2nd day of life and 79% by the 7th day, with a final total of 96% being colonised, including with *S. capitis* [80].

The most common presentation for *S. capitis* infection in neonates was bacteraemia, with or without the presence of a central venous catheter (CVC) [81]. In one CoNS bacteraemia study, 50% of cases were catheter-related, and 67.1% of these were linked to CVCs, while other sources included the digestive tract (12.1%) and skin (8.5%) [82]. In a 2020 study by Adeghate et al. [83], it was noted that gastrointestinal tract colonisation with CoNS was associated with an increased incidence of bacteraemia, with both CoNS and Enterobacterales species [83]. Risk factors for *S. capitis* bacteraemia include prior surgical treatment, receiving broad-spectrum antibiotics in the previous two weeks and longer hospital stays [84]. Colonisation with *S. capitis*, especially with the NRCS-A clone, has also been associated with an increased risk of both bacteraemia and requirement for ventilation support in neonates [85]. In a study of 105 neonates with CoNS sepsis by Ben Said et al. [86], severe morbidity was more common in neonates with *S. capitis* (55.4%) than the non-*S. capitis* group (32.0%) [86]. In some NICUs, *S. capitis* has overtaken *S. epidermidis* as the leading cause of late-onset sepsis [87]. There was limited reporting on other long-term health outcomes in neonates.

## 10. Neonatal Outbreaks

Outbreaks of *S. capitis* within healthcare environments are predominantly associated with the *S. capitis* subspecies *urealyticus*, particularly linked to the NRCS-A clone, which is known to have caused outbreaks within NICUs [88]. The NRCS-A clone has a global distribution, with isolates detected in 22 countries across Europe, the Americas, Southern Asia and Oceania [76]. Isolates show high levels of similarity, with one study demonstrating that 96% of 86 isolates identified as belonging to the NRCS-A clone from Australia, Belgium, France and the UK exhibited >80% genetic similarity [89,90]. However, it has significantly increased in prevalence since 2010, with a number of outbreaks identified within NICUs, although cases of the NRCS-A clone have been reported in an older paediatric patient and in adults [41,76,87,91]. It is estimated that a common ancestor for the NRCS-A clone emerged in the 1960s in the USA and that there was then a 10-fold increase in cases linked to this clone in the 1980s that coincided with the establishment of NICUs and increased use of vancomycin therapy [76]. There appear to be three sublineages of the NRCS-A clone, but the mutation level within the clone is low and appears to have been highly conserved since 2000 [90]. The increased prevalence of the NRCS-A clone is postulated to be linked to the levels of nonvancomycin antistaphylococcal drugs that are extensively utilised in NICUs, and the reduced sensitivity of the NRCS-A clone to the regularly used vancomycin [76].

While most infections linked to the NRCS-A clone have been associated with bacteraemia within NICUs, there have been reports of the NRCS-A clone identified in prosthetic joint infections in adults in three areas of Sweden. However, no adult outbreaks have been reported in the peer-reviewed literature [91]. Most centres in France still routinely use vancomycin to treat bacteraemia, even in the presence of an NRCS-A clone outbreak, as the isolates are phenotypically sensitive to vancomycin, although they are resistant to gentamicin and methicillin [82].

## 11. Environmental Sources and Reservoirs

Multiple studies have investigated the sources and reservoirs of *S. capitis* within hospital settings. Salimi et al. [92] showed that nursing and medical staff carried *S. capitis* in their nasal cavity as a commensal bacterium [92]. In a second *S. capitis* study, NRCS-A was identified by swabbing the hands of caregivers in an NICU, and it was hypothesised that this could be a cause of interpatient transmission [88].

Within NICU outbreak settings, environmental contamination has been noted to be of particular significance. The source of a *S. capitis* outbreak in a French NICU was traced back to contaminated almond oil bottles assigned to individual beds [93]. Multiple studies have reported isolation of *S. capitis* from the surfaces of incubators in NICUs, and a study by Cadot et al. [94] recovered bacteria from 100% of NICU mattresses (*n* = 26), with *S. capitis* isolated on 7.1% [94]. Work by Chavignon et al. [95] also found *S. capitis* on 100% of incubators (*n* = 2) in an NICU both before and after disinfection, while Butin et al. [88] isolated *S. capitis* NRCS-A in 57% (*n* = 56/99) of samples from NICU incubators [88,95].

Medical equipment has been demonstrated to be a source for bacterial surface contamination both in NICU outbreaks and in adult settings. *S. capitis* has been identified on reusable electroencephalography cup electrodes and lead wires [96], thyroid radiation shields [97] and postsurgery anaesthetic syringes [98]. Slater et al. [99] also reported *S. capitis* in 8 of 40 needleless connectors connected to peripheral IV catheters in adult medical patients in hospitals, with the authors highlighting the urgency of developing an effective needleless connector decontamination procedure [99].

*S. capitis* infection is a potential complication of surgery, and therefore surgical environments and equipment have been considered as a source of *S. capitis*. Clesham et al. [100] analysed theatre shoes (*n* = 40), finding *S. capitis* contamination on 5% (*n* = 2) [100]. A study of 101 surgical cases in Nottingham by Mahida et al. [101] showed bacterial surface contamination in 12 cases, including 2 *S. capitis*. This included contamination of 9% of IV extension lines, leading to an increased risk of bacteraemia [101].

## 12. Decontamination

In light of the links demonstrated between environmental surface contamination and transmission of *S. capitis* within clinical settings, especially within NICUs, surface decontamination is a crucial intervention. *S. capitis* is linked with biofilm production, which can be induced by environmental stressors (see Section 6); as such, a number of different decontamination approaches have been attempted to evaluate efficacy and support development of decontamination strategies.

Steam cleaning was implemented in one study as a way to disinfect heating tables and incubators and demonstrated a statistically significant reduction in loads (*p* < 0.001), both pre and post processing [102]. Another study demonstrated a reduction in *S. capitis* catheter-related bacteraemia linked to a twice-daily scrubbing routine with packed alcohol gauze of intravascular lines and hubs, saturation monitor lines and oximetry tips, electrocardiography connecting jacks, electrocardiography leads, stethoscopes and thermometers [103]. This further strengthens the association between environmental contamination and infection with *S. capitis*.

As colonisation is linked to an increased risk of bacteraemia, decontamination of patient skin could play a role in reducing infection risk, where clinical benefits are perceived to outweigh the risks. A reduction in levels of *S. capitis* colonisation has been demonstrated to be possible utilising chlorhexidine gluconate or isopropyl alcohol 70%. Taha et al. [104], however, highlighted that biofilm reduces the efficacy of skin disinfectants on blood donor skin. Furthermore, if *S. capitis* was present in biofilm, it was 64-fold more resistant than when it was in a planktonic state [104,105]. In a study by Tran et al. [106], *S. capitis* did not demonstrate any regrowth after chlorhexidine gluconate skin cleansing, although it demonstrated a 29% regrowth after isopropyl alcohol alone [106]. *S. capitis* is also more sensitive to decontamination with either isopropyl alcohol or isopropyl alcohol plus chlorhexidine gluconate when grown in mixed-species biofilm with other CoNS [104]. In general, *S. capitis* is sensitive to skin antiseptics such as chlorhexidine digluconate, benzalkonium chloride and acriflavine, although these strains carrying a QacA/B gene may have reduced susceptibility [107].

## 13. Conclusions

Invasive infections caused by *S. capitis* are an increasing clinical issue, particularly within neonatal units. In adult populations, *S. capitis* tends to cause bacteraemia and implant-associated infections rather than outbreaks. Its ability to form biofilm may play a role both in clinical presentation and environmental transmission routes associated with outbreaks within NICUs. The role of biofilm formation is key in the reduced efficacy of standard decontamination techniques and some aspects of antimicrobial treatment failures and needs to be investigated further. The mainstay of antimicrobial therapy is the use of vancomycin but use of linezolid has also been reported. Vancomycin heteroresistance is increasingly linked to the circulating NRCS-A clone; therefore, the increasing spread and detection of this clone has potential treatment implications for healthcare settings.

Although the NRCS-A clone has been detected for a number of decades, improved microbiological identification (e.g., MALDI-ToF and whole-genome sequencing) has contributed to its increased detection in NICUs and outbreak settings. The mechanism of its spread and the best interventions to mitigate it remain unclear. Additional work would help understand the prevalence of carriage in neonates outside of outbreak scenarios and the best intervention package in order to control outbreaks once they occur.

## Figures and Tables

**Table 1:** Breakdown of the categories of articles in the literature review.

Grouping	Number
Antimicrobial Resistance	81
Clinical Management Adults/General	44
Clinical Management Neonates	33
Detection	33
Environment and Decontamination	25
Genomic and Biochemical Analysis	82
Guidelines	2
Outbreak and Epidemiology	21

## Data Availability

No new data were created or analysed in this study. Data sharing is not applicable to this article.
